# Specific miRNAs Change After 3 Months of GH treatment and Contribute to Explain the Growth Response After 12 Months

**DOI:** 10.3389/fendo.2022.896640

**Published:** 2022-06-22

**Authors:** Cecilia Catellani, Gloria Ravegnini, Chiara Sartori, Beatrice Righi, Pietro Lazzeroni, Laura Bonvicini, Silvia Poluzzi, Francesca Cirillo, Barbara Predieri, Lorenzo Iughetti, Paolo Giorgi Rossi, Sabrina Angelini, Maria Elisabeth Street

**Affiliations:** ^1^Department of Mother and Child, Azienda Unità Sanitaria Locale – IRCCS di Reggio Emilia, Reggio Emilia, Italy; ^2^PhD Program in Clinical and Experimental Medicine, University of Modena and Reggio Emilia, Modena, Italy; ^3^Department of Pharmacy and Biotechnology, University of Bologna, Bologna, Italy; ^4^Epidemiology Unit, Azienda Unità Sanitaria Locale – IRCCS di Reggio Emilia, Reggio Emilia, Italy; ^5^Department of Medical and Surgical Sciences of the Mother, Children and Adults, University of Modena and Reggio Emilia, Modena, Italy; ^6^Department of Medicine and Surgery, University of Parma, Parma, Italy

**Keywords:** miR-199a-5p, miR-335-5p, miR-494-3p, growth, GH deficiency, GH treatment

## Abstract

**Context:**

There is growing evidence of the role of epigenetic regulation of growth, and miRNAs potentially play a role.

**Objective:**

The aim of this study is to identify changes in circulating miRNAs following GH treatment in subjects with isolated idiopathic GH deficiency (IIGHD) after the first 3 months of treatment, and verify whether these early changes can predict growth response.

**Design and Methods:**

The expression profiles of 384 miRNAs were analyzed in serum in 10 prepubertal patients with IIGHD (5 M, 5 F) at two time points before starting GH treatment (t−3, t0), and at 3 months on treatment (t+3). MiRNAs with a fold change (FC) >+1.5 or <-1.5 at t+3 were considered as differentially expressed. *In silico* analysis of target genes and pathways led to a validation step on 8 miRNAs in 25 patients. Clinical and biochemical parameters were collected at baseline, and at 6 and 12 months. Simple linear regression analysis and multiple stepwise linear regression models were used to explain the growth response.

**Results:**

Sixteen miRNAs were upregulated and 2 were downregulated at t+3 months. MiR-199a-5p (*p* = 0.020), miR-335-5p (*p* = 0.001), and miR-494-3p (*p* = 0.026) were confirmed to be upregulated at t+3. Changes were independent of GH peak values at testing, and levels stabilized after 12 months. The predicted growth response at 12 months was considerably improved compared with models using the common clinical and biochemical parameters.

**Conclusions:**

MiR-199a-5p, miR-335-5p, and miR-494-3p changed after 3 months of GH treatment and likely reflected both the degree of GH deficiency and the sensitivity to treatment. Furthermore, they were of considerable importance to predict growth response.

## 1 Introduction

During the last decade, knowledge on epigenetics has increased, and in this context, microRNAs (miRNAs) have attracted the interest of researchers given their role as key regulators of multiple biological processes. MiRNAs are endogenous small non-coding RNAs that act as transcriptional (1, 2) and post-transcriptional regulators (3). Multiple changes in miRNA abundance can occur, where simultaneously up- and downregulated miRNAs can target the same gene with a range of predicted effects and, *vice versa*, a single miRNA can regulate several target genes (4). To date, the miRNA network is considered of fundamental importance for the regulation of gene expression (5). MiRNAs are key regulators of metabolic pathways (6–9), and are currently studied as biomarkers of disease and response to drug administration (10, 11). Evidence on longitudinal growth regulation by miRNAs has been reported in different *in vitro* and animal models (12, 13) and miRNAs have been described to contribute to the regulation of the hypothalamic–pituitary–IGF axis and to growth plate function (12). Currently, only one study has shown that miRNAs change under conditions of dysregulated growth hormone (GH) levels in humans (14). One *in vitro* study highlighted that the GH receptor can be regulated by specific miRNAs, suggesting that this regulatory system could be of importance for the GH axis (15). Finally, one recent study described that circulating miRNAs in adult patients and mice with congenital GH deficiency were regulated in relationship with aging (16). However, so far, no studies have investigated the changes in miRNA circulating levels in response to GH treatment in childhood.

The growth response in patients on GH treatment is variable depending both on the patient’s basal conditions and on personal innate sensitivity to therapy (17). Often, the measured growth rate does not coincide with the expected one and the degree of correlation between clinical–auxological parameters and dose and GH peak vary enormously, both inter- and intra-individually during treatment. In this context, some patients run the risk of receiving an excessively low or high GH dose (18–20). Currently, in the attempt to improve the growth response, some medical devices on web platforms have become available in clinical practice (21). However, these interactive tools use universal algorithms based on growth prediction models built by collecting clinical data stored in international databases, and they can be used only after the first year of treatment.

This study aimed to identify changes in circulating miRNAs following GH treatment in children with isolated idiopathic GH deficiency (IIGHD) after the first 3 months of treatment, to explore their associations with clinical and biochemical parameters during the first 12 months of therapy, and to test the ability of early changes in these selected miRNAs to predict the clinical outcome in terms of growth on GH treatment.

## 2 Patients and Methods

### 2.1 Patients

Ten prepubertal children at diagnosis of idiopathic isolated GH deficiency were enrolled for a preliminary profiling step [chronological age (CA): 8.80 ± 2.60 years; 5 male patients (M) and 5 female patients (F)]. Twenty-five prepubertal patients were included in the following validation step (CA: 9.08 ± 3.05 years); the main characteristics of the study cohort at diagnosis are reported in **Table 1**. All subjects were diagnosed with isolated idiopathic growth hormone deficiency (IIGHD) according to the official indications (22) and remained prepubertal throughout this 12-month study. At diagnosis, 24 subjects underwent an arginine stimulation test, and 1 underwent a clonidine stimulation test as the first test. Nineteen underwent a glucagon stimulation test and six underwent a clonidine stimulation test as the second test. All the subjects underwent a magnetic resonance imaging (MRI) scan of the hypothalamus and pituitary gland. Patients were enrolled at the pediatric endocrine centers in Reggio Emilia and Modena. Patients with ascertained or probable genetic syndromes (e.g., skeletal dysplasia, Silver-Russell syndrome) and/or obesity were excluded to further reduce the chances of confounding factors. The patients were treated with GH, according to the indications of the Italian Regulatory Agency (AIFA Note 39) and underwent routine practice for treatment. Both biosimilar and recombinant human GH were used.

**Table 1:** Auxological and biochemical features of patients at baseline, and at 6 and 12 months of GH treatment.

	Baseline	6 months of treatment	12 months of treatment
Sex, M/F	17/8		
CA, years	9.08 ± 3.05		
Target height, cm	166.1 ± 8.12		
Target height SDS	−0.96 ± 0.81		
Highest GH peak N<5 ng/ml/N>5 ng/ml	7/18		
GH peak at first test, ng/ml	4.23 ± 2.06		
GH peak at second test, ng/ml	5.14 ± 2.01		
Bone age, years	7.35 ± 2.77		8.41 ± 2.88
Height, cm	119.22 ± 16.79	124.22 ± 16.88	127.96 ± 16.78
Height SDS	−1.92 ± 0.37	−1.61 ± 0.39^*^	−1.48 ± 0.39^#^
Weight, kg	23.17 ± 7.39	25.38 ± 8.51	27.39 ± 9.10
Weight SDS	−1.79 ± 0.74	−1.62 ± 0.79^*^	−1.45 ± 0.79^#^
BMI, kg/m^2^	15.85 ± 1.48	15.92 ± 1.78	16.21 ± 1.93
BMI SDS	−0.49 ± 0.79	−0.61 ± 0.88	−0.55 ± 0.91
Growth velocity, cm/year ^§^	4.49 ± 1.59	8.03 ± 1.69	7.19 ± 1.51
Growth velocity SDS ^§^	−1.60 ± 1.04	2.78 ± 1.99^*^	1.82 ± 2.40^#^
IGF-I, ng/ml	150.96 ± 62.86	281.22 ± 127.69	284.33 ± 113.92
IGF-I SDS	−0.03 ± 0.59	0.88 ± 0.76^*^	0.79 ± 0.63^#^
Fasting blood glucose, mg/dl	81.44 ± 4.75	85.44 ± 8.61	86.07 ± 7.14
Insulin, µU/ml		7.73 ± 4.41	8.83 ± 5.30
HbA1c, mmol/mol		33.18 ± 3.00	32.83 ± 2.96
Alkaline phosphatase, U/L	267.43 ± 136.97	603.59 ± 216.75	448.33 ± 227.44
Drug, N recombinant/N biosimilar	11/14		
GH dose, mg/kg/day	0.028 ± 0.004	0.023 ± 0.004	0.023 ± 0.004
Hypothalamus–pituitary MRI	*N* = 1 Rathke’s cleft cyst *N* = 2 small pituitary gland *N* = 22 normal		

BMI, body mass index; CA, chronological age; F, females; FBG, fasting blood glucose; GH, growth hormone; HbA1c, glycated hemoglobin; IGF-I, insulin-like growth factor 1; M, males; N, number; SD, standard deviation; SDS, standard deviation score; U, units. ^§^calculated on the previous 6 months. *p < 0.0001 baseline vs. 6 months on treatment. ^#^p < 0.0001, baseline vs. 12 months on treatment. Data are reported as mean ± SD.

For the purpose of analyses, patients were also subdivided according to GH peak concentrations (highest peak > or < 5 ng/ml) at testing, and based on response to GH treatment [change in height (Ht) SDS after 12 months on treatment > or < +0.3 SDS, defined as responders and non-responders, respectively) (23).

The study was approved by the local Ethical Committee (Study title: “Role of miRNAs as predictors of response to growth hormone (GH) in patients with GH deficiency” Prot no. 2016/0002409) at the Institutions. Written informed consent was obtained from all participants and their parents as appropriate.

**Figure 1:**
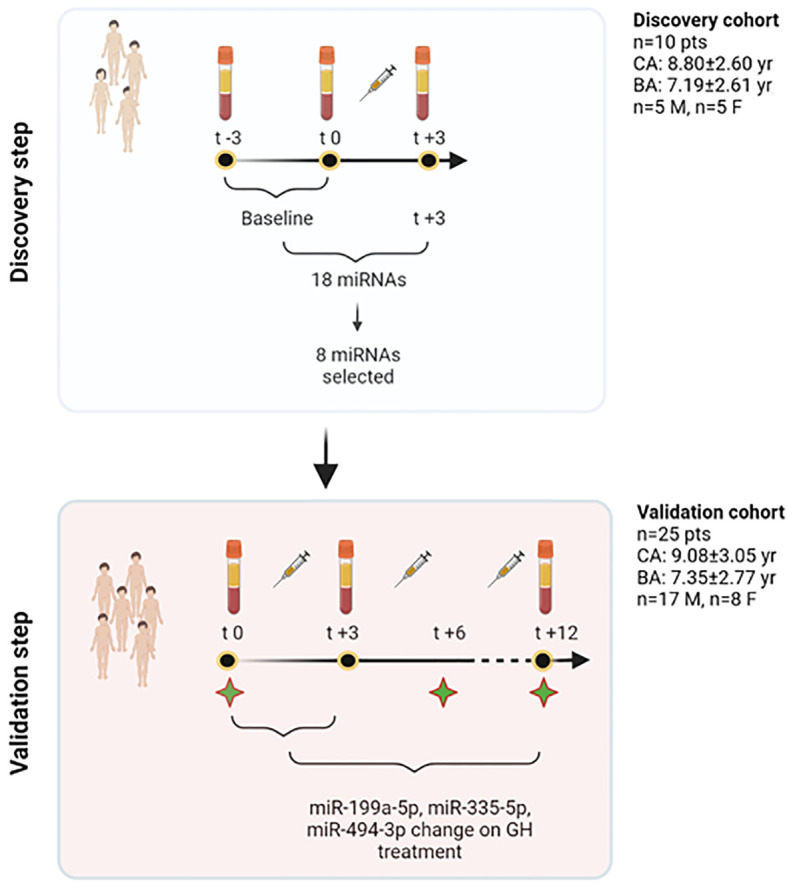
Study workflow. In the discovery step, a profiling approach was used, and all those miRNAs showing spontaneous variations independent of treatment (−3 and 0 months) were excluded from further analyses. Eighteen miRNAs were found to show changes at +3 months. Based on their function, 8 miRNAs were selected out of this initial pool of 18. The validation phase used TaqMan Real-Time qRT-PCR approach and studied the response of these 8 miRNAs at 3 months on GH treatment in 25 patients. Three specific miRNAs were found to change significantly on treatment, and were included, together with other clinical and biochemical variables, to contribute to explain growth after 1 year of treatment. To further understand miRNA changes on treatment, these were further measured in serum at 12 months. Green stars in the figure represent time points in which clinical and biochemical data have been recorded (t0, t+6, t+12). BA, bone age; CA, chronological age; F, females; GH, growth hormone; M, males; n, number; pts, patients; yr, years. Created with BioRender.com.

The general workflow of the study is described in **Figure 1**.

### 2.2 Sample Processing and Total RNA Isolation

Whole blood was drawn in BD Vacutainer Serum Separator Tubes, and it was processed within 2 h from collection and after overnight fasting, between 7:30 and 8:30 a.m. Whole blood was then centrifuged at 2,000 *g* for 10 min at 4°C. Serum was aliquoted in 1.5-ml sterile RNase-free tubes and further centrifuged at 2,500 *g* for 10 min at 4°C to remove any contaminant cells and debris. Serum was then collected in sterile RNase-free tubes and stored at −80°C until use. Blood samples were collected at two time points before the beginning of treatment (3 months before, t−3, and just before the treatment, t0), and at 3 and 12 months after the beginning of the treatment (t+3 and t+12) in the context of routine controls. Total RNA was isolated from 400 μl of serum using the miRVana PARIS kit (Invitrogen Cat No. AM1556) according to the manufacturer’s protocol and the eluate was stored at −80°C. RNA was reverse-transcribed using the TaqMan™ Advanced miRNA cDNA Synthesis Kit (Applied Biosystems Cat No. A28007) following the manufacturer’s instructions.

### 2.3 Discovery Step: miRNA Expression Profiling

The expression profiles of 384 miRNAs were analyzed in 10 patients, of which 5 were male patients and 5 were female patients (10 samples collected at t−3, 10 samples at t0, and 10 samples at t+3) to avoid gender-specific miRNAs, using TaqMan Advanced miRNA Human A Cards (Applied Biosystems Cat No. A34714) that contain 384 miRNA assays. Briefly, 2 μl of RNA eluate was reverse-transcribed to cDNA, using the TaqMan Advanced miRNA cDNA synthesis kit (Applied Biosystems Cat No. A28007) following the manufacturer’s instructions in the Thermal Cycler T100 (Bio-Rad). The cDNA was loaded into the TaqMan Advanced miRNA array Card A and run in a 7900HT Fast PCR system (Applied Biosystems). Array data were normalized using the standard internal reference hsa-miR-16-5p (Assay ID: 477860_mir) as endogenous control (24); to be sure about its validity, we selected hsa-miR-16-5p after assessment of its stability in our study cohort and further review of the literature (24). The data were analyzed using the 2^−ΔΔCt^ method and miRNAs with Ct > 35 were considered as not expressed and excluded from further analysis. Moreover, those miRNAs changing significantly (*p*-value at paired Student’s *t*-test ≤0.05) in the two time points before starting treatment (t−3 and t0) were excluded in order to not consider those miRNAs changing for other causes independent of treatment. Considering the exploratory purpose of the study and based on the number of subjects analyzed, we did not perform FDR correction. The miRNAs having a fold change (FC) (log_2_2^−ΔΔCt^) >+1.5 or FC (log_2_2^−ΔΔCt^) < −1.5 between baseline and 3 months on treatment were considered as differentially expressed.

An *in silico* analysis was performed to identify the validated target genes and pathways for each differentially expressed miRNA in order to select those expected to be involved with growth. In particular, the network that represents miRNA target interactions and highlights the most impacted pathways was obtained from the miRNet v 2.0 online tool (25). This tool collects data from three well-annotated databases, miRTarBase v8.0, TarBase v8.0, and miRecords. The significance was set at a *p*-value of 0.05.

### 2.4 Validation of the Profiling Results by Real-Time qRT-PCR

MiRNAs for the validation step were chosen based on FC (>+1.5 or <−1.5) between baseline and 3 months on treatment, and based on miRNA target gene analysis. Eight miRNAs were selected and evaluated by TaqMan Advanced miRNA assays (Applied Biosystems): hsa-miR-22-3p (Assay ID: 477985_mir), hsa-miR-30c-5p (Assay ID: 478008_mir), hsa-miR-106a-5p (Assay ID: 478225_mir), hsa-miR-140-5p (Assay ID: 477909_mir), hsa-miR-199a-5p (Assay ID: 478231_mir), hsa-miR-335-5p (Assay ID: 478324_mir), hsa-miR-340-5p (Assay ID: 478042_mir), and hsa-miR-494-3p (Assay ID: 478135_mir). cDNA was prepared as described above (*Section 2.3*), and miRNA expression was evaluated according to the manufacturer’s protocol and run in triplicate in a 7900HT Fast PCR system (Applied Biosystems). Data were normalized using hsa-miR-16-5p (Assay ID: 477860_mir) as endogenous control (24). The validation analysis was performed at two time points t0 and t+3. In addition, miRNA levels were analyzed at 12 months on treatment (t+12).

### 2.5 Auxological Parameters and Bone Age

A full auxological assessment was done at baseline, 6 months, and 12 months on treatment and medical history was taken. Body mass index (BMI), calculated as weight/height^2^ (kg/m^2^), and weight were converted to standard deviation scores (SDS) using the references of Cole et al. (26). Height (Ht) and target height (THt) were recorded and expressed as SDS, using the Tanner reference data (27). Height velocity SDS was calculated using Tanner’s references (27). Bone age was assessed according to the Greulich and Pyle Atlas reference (28). Puberty in all subjects was staged according to the criteria of Marshall and Tanner (29, 30).

### 2.6 Biochemical Parameters

Alkaline phosphatase and blood glucose were measured using Atellica CH Alkaline Phosphatase Concentrated (ref. 11097600) and Atellica CH Glucose Hexokinase 3 (ref. 11097592) assays on the Atellica CH Analyzer, respectively. HbA1c was assayed by HPLC on the D-100 System (Bio-Rad). Insulin, GH, and IGF-I levels were measured using chemiluminescence methods: LIAISON Insulin (Ref. 310360), LIAISON hGH (ref. 310340), and LIAISON IGF-I (ref. 313231) immunoassays, respectively, on the LIAISON Analyzer Diasorin. IGF-I concentrations were converted to SDS based on the reference values provided by the manufacturer.

### 2.7 Statistical Analysis

Paired Student’s *t*-test (*p* ≤ 0.05) was used to identify miRNAs that were differentially expressed after 3 months of GH treatment with respect to the baseline (t0, t+3). Moreover, paired Student’s *t*-test was used to evaluate differences between baseline patient’s characteristics and after 6 and 12 months of treatment. These comparisons were made only on the standardized parameters.

**Figure 2:**
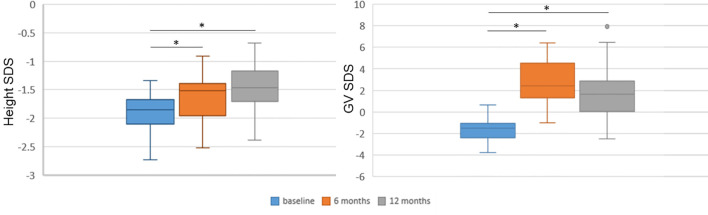
Height and growth velocity over time. Height and growth velocity are represented with respect to the previous 6 months. Height and growth velocity (GV) were standardized according to Tanner’s standards. SDS, standard deviation score. *p < 0.0001 versus baseline at paired Student’s *t*-test.

The associations of miRNA values at baseline, at 3 months and their change during this time frame (delta 0–3 months) with baseline clinical features, and with 6- and 12-month recorded clinical and biochemical data were analyzed. In addition, the associations of 12-month miRNA levels with clinical and biochemical parameters at 12 months were investigated by means of simple linear regression analysis. The distribution of height SDS and growth rate SDS at baseline and at 6 and 12 months were also analyzed (**Figure 2**) in order to select the meaningful time horizon for measuring the two outcomes.

Multiple linear regression models were performed in order to investigate the major determinants of height variations between 0 and 6 months and between 0 and 12 months, and the variance of growth rate variation between 0 and 6 months. A set of models that considered only auxological parameters at baseline were estimated: the first one included only auxological parameters; the other models were estimated by adding, one by one, as independent variables, GH peaks at testing, GH dose at the beginning of treatment, IGF-I SDS at baseline, the difference between CA and bone age at baseline, and baseline levels and change in miRNA levels during the first 3 months of treatment. Finally, the models were estimated with a backward stepwise selection estimation method with significance level for removal from the model set to 0.2 considering the following as independent variables: sex, CA at the beginning of treatment, genetic target SDS, treatment dose (mg/kg/day), height SDS at baseline, weight SDS at baseline, peak at first GH stimulation test (ng/ml), peak at second GH simulation test (ng/ml), IGF-I SDS (t0), difference between CA and bone age at baseline, miR-199a-5p (t0), delta (0–3) miR-199a-5p, miR-355-5p (t0), delta (0–3) miR-335-5p, miR-494-3p (t0), and delta (0–3) miR-494-3p.

To define the degree of accuracy or predictive effectiveness of the model, leave-one-out cross-validation analysis was performed for each model. Specifically, we reported the variations in adjusted *R*^2^, meaning the percentage of variance in the outcome explained by the variable included in the model adjusted for the number of degrees of freedom spent by the model parameters, and *R*^2^ CV as a measure of the variance, explained by the models corrected with the cross-validation method. In this way, we built 25 models excluding patients one by one, and we predicted the growth parameter of the excluded patient with the parameters estimated by the model. The best models obtained are reported in the text. For the associations explored in these models, we did not perform a formal statistical test of hypothesis, and *p*-values should be interpreted as continuous variables without any threshold. Furthermore, *p*-values should be interpreted cautiously because there is an issue of multiple testing, but no easy solutions for false discovery rate adjustments were identified since it is not possible to determine the total set of independent comparisons. Comparison of miRNA expression levels in responders vs. non responders was also performed, as well as a comparison according to GH peak concentrations as specified above. Statistical analyses were performed using STATA v16.0 (STATA Corp., College Station, TX, USA).

## 3 Results

### 3.1 Growth and Biochemical Outcomes During the First Year of Treatment

All clinical and biochemical characteristics of the subjects are summarized in **Table 1**. Height SDS both at 6 months and at 1 months on treatment was significantly increased with respect to baseline (-1.92 ± 0.37 vs. −1.61 ± 0.39, *p* < 0.0001, and −1.92 ± 0.37 vs. −1.48 ± 0.39, *p* < 0.0001, respectively) (**Figure 2**). Although BMI SDS decreased on treatment, no significant change was observed. Growth velocity (GV) SDS after 6 months (−1.60 ± 1.04 vs. 2.78 ± 1.99, *p* < 0.0001) and 12 months on treatment was significantly increased with respect to pre-treatment growth velocity, but decreased during the second 6 months on GH (2.78 ± 1.99 vs. 1.82 ± 2.40) (**Figure 2**). IGF-I SDS increased after 6 months on treatment with respect to baseline (0.88 ± 0.76 vs. −0.03 ± 0.59, *p* < 0.0001), remaining almost stable in the following 6 months on treatment. Alkaline phosphatase increased on treatment but changes were not statistically significant, whereas fasting blood glucose and HbA1c remained stable.

### 3.2 Discovery miRNA Profiling Step

The profiling step was preliminarily performed in order to select a pool of miRNAs of interest for the following analyses. Among the miRNAs analyzed, 186 were detectable and measured in the serum of patients. Those miRNAs changing significantly (*p* < 0.05 at paired Student’s *t*-test) between the two time points before starting treatment (t−3; t0) were excluded, as changes were considered to be independent of treatment. Sixteen miRNAs (hsa-let-7a-5p, hsa-let-7e-5p, hsa-miR-30c-5p, hsa-miR-34a-5p, hsa-miR-132-3p, hsa-miR-140-5p, hsa-miR-199a-5p, hsa-miR-330-3p, hsa-miR-335-5p, hsa-miR-340-5p, hsa-miR-369-3p, hsa-miR-375, hsa-miR-450a-5p, hsa-miR-494-3p, hsa-miR-582-5p, and hsa-miR-421) had an FC >+1.5 and were considered as upregulated after 3 months of treatment with respect to baseline; two miRNAs (hsa-miR-22-3p and hsa-miR-106a-5p) had an FC < −1.5 and were considered to be downregulated after 3 months of GH treatment with respect to baseline.

### 3.3 Pathway Enrichment Analysis

**Figure 3:**
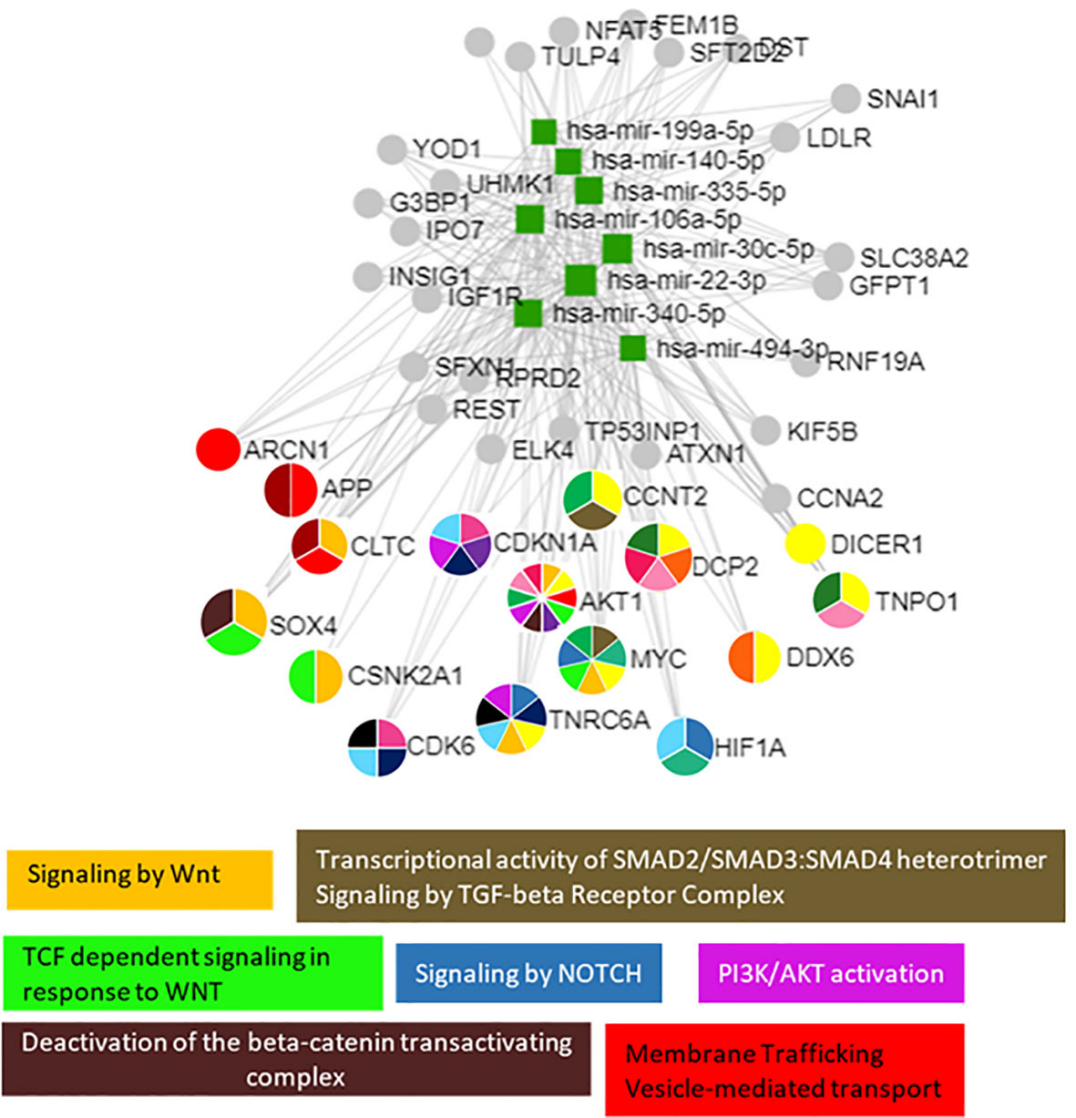
The 8 selected miRNAs regulated genes and pathways that are important for growth. Analyses were carried out using miRNet v 2.0 and only significantly predicted pathways (*p* < 0.05) were selected to generate the figure.

Pathway enrichment analysis of these 18 miRNAs evidenced that they were significantly involved in the regulation of 100 different pathways (*p* < 0.05). Among these 18 miRNAs (**Table 2**), those predicted to be involved in the regulation of longitudinal growth and bone development were selected for the validation step, namely, miR-22-3p, miR-30c-5p, miR-106a-5p, miR-140-5p, miR-199a-5p, miR-335-5p, miR-340-5p, and miR-494-3p (**Figure 3**). These miRNAs were predicted to regulate genes involved in Wnt-β-catenin signaling, Notch signaling, PI3K/AKT, and TGF-β signaling that are relevant for growth (**Figure 3**).

**Table 2:** Differentially expressed miRNAs after 3 months of GH treatment with respect to the baseline in 10 prepubertal patients with IIGHD.

miRNA	Fold Change (log_2_2^−ΔΔCt^)	Up/down
hsa-miR-375	3.1	Up
hsa-let-7e-5p	2.5	Up
hsa-miR-340-5p	2.4	Up
hsa-miR-494-3p	2.1	Up
hsa-miR-34a-5p	2.1	Up
hsa-miR-30c-5p	1.9	Up
hsa-let-7a-5p	1.8	Up
hsa-miR-140-5p	1.8	Up
hsa-miR-421	1.8	Up
hsa-miR-132-3p	1.7	Up
hsa-miR-330-3p	1.7	Up
hsa-miR-369-3p	1.7	Up
hsa-miR-582-5p	1.7	Up
hsa-miR-450a-5p	1.6	Up
hsa-miR-335-5p	1.6	Up
hsa-miR-199a-5p	1.6	Up
hsa-miR-106a-5p	−1.8	Down
hsa-miR-22-3p	−3.1	Down

Fold change (FC) was calculated as log_2_2^−ΔΔCt^. MiRNAs with an FC > 1.5 were considered as upregulated and miRNAs with an FC < −1.5 were considered as downregulated.

### 3.4 miRNA Validation Step

**Figure 4:**
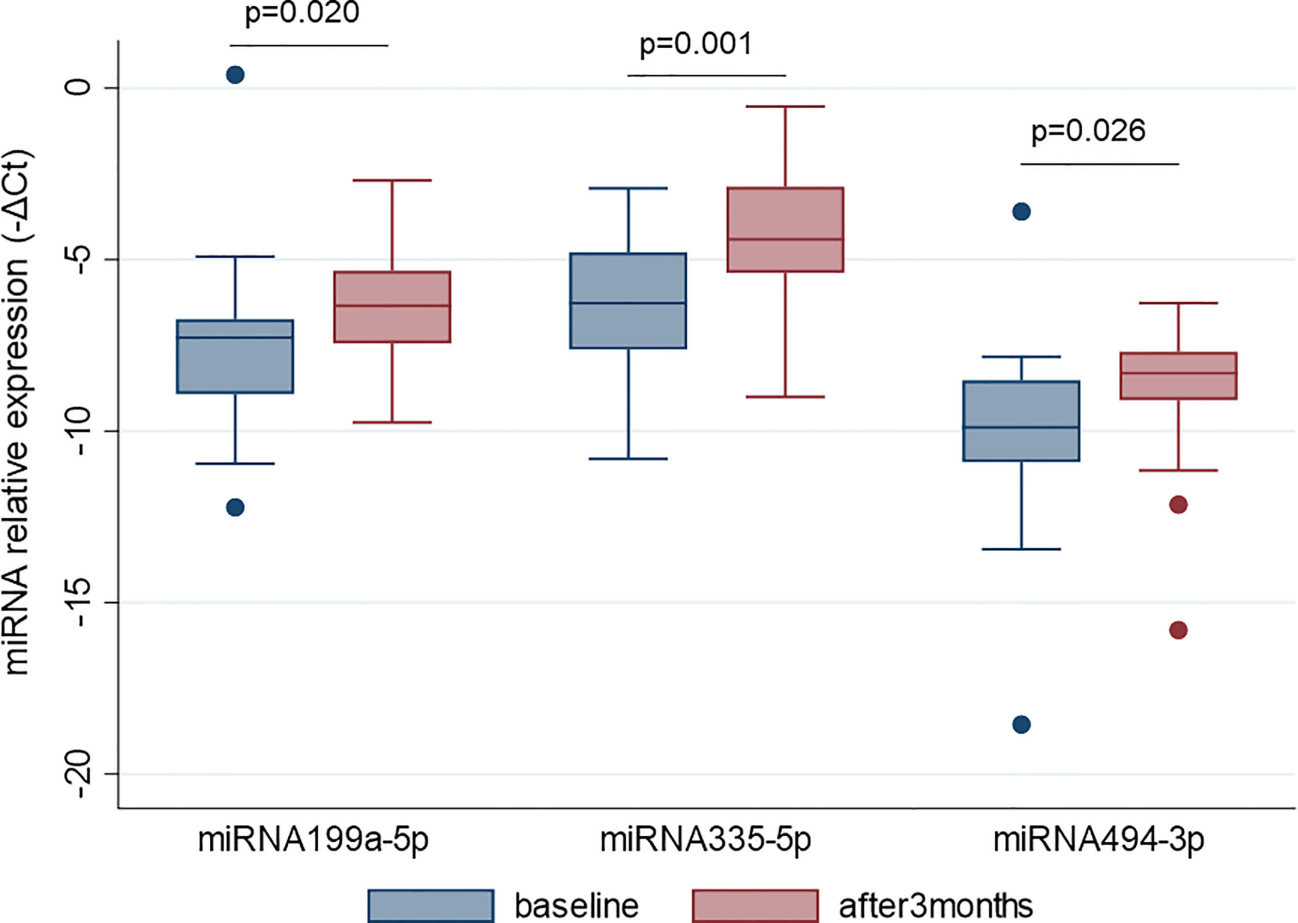
MiR-199a-5p, miR-335-5p and miR-494-3p are upregulated after 3 months of GH treatment with respect to the baseline. MiRNA levels are expressed as –ΔCt where ΔCt is calculated as: Ct miRNA − Ct miR-16-5p. *p*-value ≤ 0.05 at paired Student’s *t*-test.

**Figure 5:**
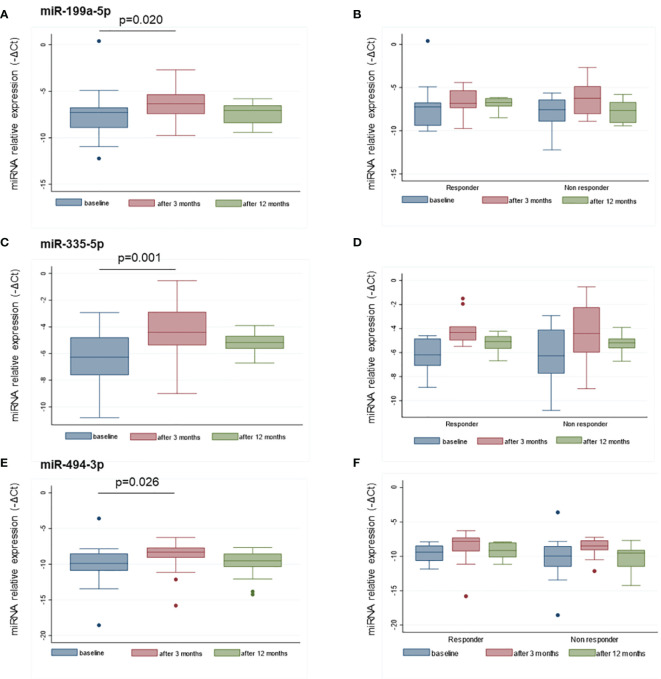
Distributions of miR-199a-5p **(A)**, miR-335-5p **(C)**, and miR-494-3p **(E)** at baseline, and after 3 and 12 months of treatment. The distribution of the same miRNAs in responders and non-responders to GH treatment are reported at the same time points in panels **(B, D, F)**. MiRNA levels are expressed as –ΔCt where ΔCt is calculated as: Ct miRNA − Ct miR-16-5p. *p*-value ≤ 0.05 at paired Student’s *t*-test.

MiR-22-3p, miR-30c-5p, miR-106a-5p, miR-140-5p, miR-199a-5p, miR-335-5p, miR-340-5p, and miR-494-3p were selected to be validated in single assay, at baseline, and at 3 months on treatment, in the larger group of patients (**Table 2**). This study highlighted that miR-199a-5p (*p* = 0.020), miR-335-5p (*p* = 0.001), and miR-494-3p (*p* = 0.026) were upregulated after 3 months with respect to baseline (**Figure 4**). The measurements at 12 months of treatment showed that the circulating levels of these specific miRNAs were more stable and that the inter-individual variance was smaller than that observed at the other two time points (**Figures 5A, C, E**, **S1**).

Based on the GH peaks (highest peak > or < 5 ng/ml), no differences in miRNA levels were observed at baseline or at 3 and 12 months, nor was there any difference in the change at 3 months versus baseline. MiRNA levels were more variable in the non-responder patients with respect to responders (< or > +0.3 SDS) (**Figures 5B, D, F**). No differences in miRNA levels were observed based on the type of GH used for treatment (biosimilar vs. rhGH).

### 3.5 Simple Linear Regression Analysis and Multiple Stepwise Linear Regression Models to Explain Growth Response

All possible associations between baseline characteristics and miRNA values at baseline, at 3 months of treatment, and miRNA variations during the first 3 months (ΔΔCt 0–3 months) were analyzed in order to evaluate whether a variable could be critical for the building of a model (**Tables 3A–C**) . Several models starting from auxological parameters at baseline, GH peaks at testing, and GH dose at the beginning of treatment, IGF-I SDS at baseline, bone age and difference between CA and bone age, baseline levels, and change in miRNA levels during the first 3 months of treatment were used to explain the change in height SDS over the first 6 and 12 months of treatment, and growth velocity during the first 6 months of treatment (**Tables 4A–C**). Change in growth velocity from 0 to 12 months after treatment was not included, since the change in growth velocity was not constant during the period as shown in **Figure 2**.

**Table 3:** Associations between baseline characteristics, miR-199a-5p **(A),** miR-335-5p **(B),** and miR-494-3p **(C)** levels at baseline, their change between baseline and 3 months (delta), and after 3 months of treatment.

A: miRNA-199a-5p																			
Baseline characteristics	miRNA-199a-5p																		
	Baseline						delta 0–3 months								3 months				
	coef	*p*-value		95% CI			coef		*p*-value		95% CI				coef	*p*-value		95% CI	
**Sex, M/F**	0.49	0.646		−1.68	2.65		−0.72		0.503		−2.89		1.46		−0.23	0.767		−1.80	1.34
**Bone age, years**	0.06	0.765		−0.35	0.46		−0.18		0.368		−0.58		0.23		−0.12	0.388		−0.40	0.16
**CA, years**	0.03	0.876		−0.31	0.36		−0.15		0.361		−0.49		0.18		−0.13	0.288		−0.37	0.11
**Height SDS**	3.34	0.009		0.91	5.78		−3.20		0.015		−5.71		−0.70		0.14	0.891		−1.91	2.19
**Weight SDS**	0.81	0.222		−0.53	2.16		−0.28		0.687		−1.67		1.12		0.54	0.266		−0.44	1.52
**BMI SDS**	0.38	0.545		−0.91	1.67		0.37		0.567		−0.94		1.67		0.75	0.093		−0.14	1.64
**IGF-I SDS**	1.42	0.088		−0.23	3.06		−0.70		0.415		−2.45		1.05		0.72	0.242		−0.52	1.95
**Alkaline phosphatase, U/L**	0.01	0.136		−0.00	0.02		−0.00		0.936		−0.02		0.02		0.01	0.108		−0.00	0.02
**GH peak at first stimulation test, ng/ml**	−0.14	0.573		−0.64	0.36		0.12		0.616		−0.38		0.63		−0.01	0.938		−0.38	0.35
**GH peak at second stimulation test, ng/ml**	−0.11	0.666		−0.62	0.40		0.12		0.640		−0.40		0.64		0.01	0.955		−0.36	0.38
**Biosimilar vs. human recombinant GH**	−0.24	0.807		−2.28	1.80		−0.00		0.999		−2.07		2.07		−0.24	0.737		−1.72	1.23
**GH dose, mg/kg/day**	34.16	0.796		−236.45	−304.78		24.37		0.856		−249.54		298.28		58.53	0.54		−136.05	253.12
**B: miRNA-335-5p**																			
**Baseline characteristics**	**miRNA-335-5p**																		
	**Baseline**						**Delta 0–3 months**								**3 months**				
	**coef**	***p*-value**		**95% CI**			**coef**			***p*-value**	**95% CI**				**coef**	***p*-value**		**95% CI**	
**Sex, M/F**	0.97	0.309		−0.96	2.91		−0.48			0.699	−2.99		2.04		0.50	0.592		−1.39	2.39
**Bone age, years**	0.22	0.200		−0.13	0.57		−0.12			0.551	−0.54		0.29		0.10	0.520		−0.22	0.42
**CA, years**	0.13	0.371		−0.17	0.44		−0.04			0.827	−0.44		0.35		0.09	0.525		−0.20	0.39
**Height SDS**	−0.07	0.954		−2.65	2.50		0.21			0.896	−3.07		3.49		0.14	0.909		−2.34	2.61
**Weight SDS**	0.31	0.611		−0.94	1.57		0.55			0.486	−1.05		2.14		−0.86	0.138		−2.02	0.30
**BMI SDS**	0.34	0.554		−0.83	1.52		0.46			0.529	−1.04		1.96		0.80	0.139		−0.28	1.89
**IGF-I SDS**	−1.26	0.097		−2.76	0.25		1.12			0.254	−0.86		3.1		−0.14	0.854		−1.67	1.40
**Alkaline phosphatase, U/L**	0.00	0.856		−0.02	0.02		0.01			0.253	−0.01		0.03		0.01	0.264		−0.01	0.03
**GH peak at first stimulation test, ng/ml**	−0.04	0.863		−0.50	0.42		−0.03			0.903	−0.62		0.55		−0.07	0.733		−0.51	0.37
**GH peak at second stimulation test, ng/ml**	0.24	0.413		−0.35	0.83		0.24			0.413	−0.35		0.83		0.23	0.296		−0.21	0.67
**Biosimilar vs. human recombinant GH**	0.98	0.276		−0.83	2.79		−0.96			0.403	−3.3		1.37		0.02	0.985		−1.77	1.80
**GH dose, mg/kg/day**	87.36	0.466		−156.39	331.11		−215.9			0.151	−516.31		84.51		−128.54	0.260		−358.94	101.86
**C: miRNA-494-3p**																			
**Baseline characteristics**	**miRNA-494-3p**																		
	**Baseline**							**Delta 0–3 months**							**3 months**				
	**coef**	***p*-value**	**95% CI**					**coef**		***p*-value**		**95% CI**			**coef**		***p*-value**	**95% CI**	
**Sex, M/F**	0.08	0.942	−2.27			2.44		−1.65		0.142		−3.90		0.60	−1.57		0.066	−3.25	0.11
**Bone age, years**	−0.31	0.141	−0.72			0.11		0.31		0.138		−0.11		0.72	−0.00		0.993	−0.34	0.34
**CA, years**	−0.24	0.168	−0.59			0.11		0.18		0.318		−0.18		0.54	−0.06		0.638	−0.35	0.22
**Height SDS**	0.36	0.808	−2.70			3.43		−2.20		0.134		−5.12		0.73	−1.83		0.102	−4.05	0.39
**Weight SDS**	0.83	0.249	−0.63			2.30		−0.78		0.282		−2.25		0.69	0.05		0.927	−1.10	1.21
**BMI SDS**	1.17	0.078	−0.14			2.49		−0.75		0.272		−2.12		0.63	0.42		0.420	−0.64	1.49
**IGF-I SDS**	2.14	0.014	0.47			3.80		−1.66		0.065		−3.42		0.11	0.48		0.499	−0.97	1.93
**Alkaline phosphatase, U/L**	0.01	0.273	−0.01			0.04		−0.01		0.491		−0.03		0.02	0.01		0.058	−0.00	0.01
**GH peak at first stimulation test, ng/ml**	0.18	0.503	−0.36			0.72		0.03		0.903		−0.51		0.58	0.21		0.299	−0.20	0.62
**GH peak at second stimulation test, ng/ml**	−0.01	0.977	−0.57			0.55		−0.01		0.971		−0.57		0.55	−0.02		0.932	−0.45	0.41
**Biosimilar vs. human recombinant GH**	−1.15	0.281	−3.31			1.01		0.24		0.822		−1.97		2.46	−0.91		0.268	−2.56	0.75
**GH dose, mg/kg/day**	−95.24	0.505	−385.97			195.49		50.98		0.722		−242.33		344.3	−44.25		0.688	−269.2	180.69

Data are presented as linear regression coefficients, *p*-value, and 95% CI. BMI, body mass index; CA, chronological age; CI, confidence interval; coef, coefficient; F, females; GH, growth hormone; IGF-I, insulin-like growth factor 1; M, males; SDS, standard deviation score. U, units.

**Table 4:** Multiple regression models to predict the change in height SDS during the first 6 months **(A)** and 12 months **(B)** of treatment, and growth velocity during the first 6 months of treatment **(C)**.

A				
		Delta height 0–6		
		Adj *R*^2^		*R*^2^ CV
**Model 1**	Sex, CA (years), GH dose (mg/kg/day), height (SDS), weight (SDS), target height (SDS)	0.53		0.11
**Model 2**	Sex, CA (years) GH dose (mg/kg/day), height (SDS), weight (SDS), target height (SDS), GH peaks (ng/ml)	0.49		0.04
**Model 3**	Sex, CA (years), GH dose (mg/kg/day), height (SDS), weight (SDS), target height (SDS), IGF-I (SDS)	0.51		0.08
**Model 4**	Sex, CA (years), GH dose (mg/kg/day), height (SDS), weight (SDS), target height (SDS), difference between CA and bone age	0.62		0.21
**Model 5**	Sex, CA (years), GH dose (mg/kg/day), height (SDS), weight (SDS), target height (SDS) and:			
	miR-199a-5p (baseline and delta 0–3)	0.59		0.15
	miR-355-5p (baseline and delta 0–3)	0.49		0.07
	miR-494 -3p (baseline and delta 0–3)	0.48		0.06
**Model 6—STEPWISE**	Selected variables: sex, CA (years), target height (SDS), GH dose (mg/kg/day), height (SDS), weight (SDS), difference between CA and bone age (years), GH peak at second test (ng/ml), miR-335-5p baseline, delta 0–3 months miR-335-5p, delta 0–3 months miR-494-3p.	0.72		0.02
**B**				
			**Delta height 0–12**	
			**Adj *R*^2^**	***R*^2^ CV**
**Model 1**	Sex, CA (years), GH dose (mg/kg/day), height (SDS), weight (SDS), target height (SDS)		0.36	0.15
**Model 2**	Sex, CA (years), GH dose (mg/kg/day), height (SDS), weight (SDS), target height (SDS), GH peaks (ng/ml)		0.31	0.02
**Model 3**	Sex, CA (years), GH dose (mg/kg/day), height (SDS), weight (SDS), target height (SDS), IGF-I (SDS)		0.36	0.10
**Model 4**	Sex, CA (years), GH dose (mg/kg/day), height (SDS), weight (SDS), target height (SDS), difference between chronological age and bone age		0.67	0.44
**Model 5**	Sex, CA (years), GH dose (mg/kg/day), height (SDS), weight (SDS), target height (SDS), and:			
	miR-199a-5p (baseline and delta 0–3)		0.41	0.20
	miR-355-5p (baseline and delta 0–3)		0.34	0.01
	miR-494 -3p (baseline and delta 0–3)		0.29	0.12
**Model 6—STEPWISE**	Selected variables: sex, CA (years), target height (SDS), GH dose (mg/kg/day), difference between CA and bone age, delta 0–3 months miR-335-5p, delta 0–3 months miR-494-3p.		0.79	0.43
**C**				
			**Delta growth rate 0–6**	
			**Adj *R*^2^**	***R*^2^ CV**
**Model 1**	Sex, CA (years), GH dose (mg/kg/day), height (SDS), weight (SDS), target height (SDS)		−0.12	0.07
**Model 2**	Sex, CA (years) GH dose (mg/kg/day), height (SDS), weight (SDS), target height (SDS), GH peaks (ng/ml)		−0.23	0.14
**Model 3**	Sex, CA (years), GH dose (mg/kg/day), height (SDS), weight (SDS), target height (SDS), IGF-I (SDS)		−0.17	0.08
**Model 4**	Sex, CA (years), GH dose (mg/kg/day), height (SDS), weight (SDS), target height (SDS), difference between CA and bone age		0.00	0.00
**Model 5**	Sex, CA (years), GH dose (mg/kg/day), height (SDS), weight (SDS), target height (SDS) and:			
	miR-199a-5p (baseline and delta 0–3)		0.39	0.23
	miR-355-5p (baseline and delta 0–3)		0.02	0.00
	miR-494 -3p (baseline and delta 0–3)		−0.05	0.01
**Model 6—STEPWISE**	Selected variables: sex, CA (years), GH dose (mg/kg/day), target height (SDS), weight (SDS), difference between CA and bone age, GH peak at first test (ng/ml), GH peak at second test (ng/ml), miR-199a- 5p baseline, delta 0–3 months miR-335-5p, delta 0–3 months miR-199a-5p.		0.75	0.63

Adj, adjusted; CA, chronological age; CV, cross-validated; GH, growth hormone; IGF-I, insulin-like growth factor 1; SDS, standard deviation score.

With regard to the change in HtSDS (0–6 months), each single miRNA gave a contribution to the model comparable to that given by IGF-I SDS and the peak GH value, without a substantial improvement in the model that used auxological parameters only. The difference between chronological age and bone age substantially increased the ability of the model to predict the outcome. The final model, obtained with a stepwise procedure, included miR-335-5p and miR-494-3p together with the other variables (**Table 4A**).

Considering the outcome delta HtSDS (0–12 months), miR-199a-5p was the miRNA that most improved the model containing the auxological parameters only, and its contribution was more important than that of IGF-I SDS and peak GH values. The difference between chronological age and bone age was the single best predictor, whereas the stepwise procedure identified a model including miR-335-5p, miR-494-3p, sex, CA, target height SDS, GH dose, and the difference between chronological age and bone age as the best highly predictive model (**Table 4B**).

With regard to growth velocity SDS (0–6 months), only one miRNA (miR-199a-5p) contributed to the model when the miRNAs were included one by one. The variance was explained better by this miRNA than by IGF-I SDS at baseline, GH peak values, and the difference between chronological age and bone age. The stepwise procedure identified a highly predictive model including miR-335-5p, miR-199a-5p, sex, CA, weight SDS, target height SDS, GH dose, peak GH values at testing, and the difference between chronological age and bone age (**Table 4C**).

## 4 Discussion

Using an miRNA profiling approach, 18 miRNAs were found to change after the first 3 months of GH treatment in IIGHD prepubertal patients. The subsequent validation phase in a larger group of patients showed that miR-335-5p, miR-199a-5p, and miR-494-3p were significantly upregulated at 3 months, and both the baseline circulating levels and their change (0–3 months) contributed to explain growth at 12 months of treatment improving the growth prediction substantially based on baseline clinical features, and GH peaks during the stimulation tests at diagnosis.

To the best of our knowledge, this is the first study that analyzes the change in circulating miRNA levels in response to GH treatment. This study considered only prepubertal subjects with IIGHD in order to reduce confounding factors. In fact, as the miRNA network is a key modulator of gene expression, it changes throughout life (13, 31, 32), and is also dependent on body weight (33).

A previous paper by Kelly et al. (15) considered miRNA expression levels as potential markers of GH administration in humans to evaluate whether they could detect doping in sports. In particular, this study involved a total of 20 subjects subdivided into three groups (6 individuals receiving replacement doses of GH, 11 acromegalic patients, and 3 individuals with no abnormalities in GH secretion); miRNA microarray analyses were performed on a subgroup of 4 acromegalic patients, 3 rhGH users, and 2 individuals with no known pituitary disorders evidencing that miR-2861, miR-663, miR-3152, and miR-3185 were reduced when rhGH was administered (15). A previous *in vitro* study reported that the GH receptor was regulated by specific miRNAs that inhibited GH receptor expression (16), suggesting that this regulatory system was of importance for the GH axis. We recently reviewed the regulation by miRNAs not only at the GH receptor level but also on the GH signaling pathway, on IGFs, and IGF1 receptor signaling in different *in vitro* and animal models highlighting their importance for growth (13). Interestingly, the miRNAs we found to be differentially expressed in the current study have not been investigated previously in the context of GH secretion, signaling, and the IGF system. These findings suggest that further relationships between miRNAs, the GH/IGF-1 axis, and the IGF system will be disclosed in the near future. With regard to the function of the specific miRNAs, miR-199a-5p and miR-335-5p, they play a role in bone formation and osteoblast differentiation *in vitro*. In particular, miR-199a-5p is involved in osteoblast differentiation, and its upregulation increases alkaline phosphatase (ALP) activity, calcification, and the expression of osteoblast differentiation markers such as Runx2, Osterix, and Osteocalcin in human bone marrow stem cells (34). The overexpression of miR-335-5p has been reported to promote bone formation and regeneration in a transgenic mouse model (35). A previous study from this same group highlighted that miR-335-5p reduced the expression of DKK1, an inhibitor of the Wnt signaling pathway, which is pivotal for bone development (36). Moreover, miR-335-5p overexpression has been shown to promote chondrogenic differentiation of mesenchymal stem cells (37). MiR-494-3p has not been studied yet in the context of bone or growth plate development; however, it has been reported to promote PI3K/AKT pathway hyperactivation in hepatocellular carcinoma by targeting PTEN (38). The PI3K/AKT pathway is known to control hypertrophic chondrocyte differentiation and to be involved in endochondral bone growth (39), and promotes osteoblast differentiation (40).

Our findings also suggest that early changes in these three specific miRNAs could reflect both sensitivity to GH treatment as well as the degree of GH deficiency at diagnosis. In support of this, we observed little difference in the levels among single subjects after 12 months of treatment, whereas a clear change occurred during the first 3 months that could depend both on the initial degree of GH deficiency and on the sensitivity to the GH being administered for treatment: in fact, different doses are often needed in different subjects. Interestingly, in the growth prediction models, the GH dose was selected among the variables that gave substantial contribution to the explanation of variance in growth. The changes in the miRNA levels were found to be independent of GH peak levels in response to stimulation tests, possibly confirming that GH peaks do not reflect the degree of GH deficiency, consistent with the well-known fact that the response to tests is variable (41). Furthermore, the degree of response to treatment did not seem to affect their change during the first 3 months of treatment.

Finally, in our series, these miRNAs proved to considerably increase the capacity to predict growth response compared with the use of clinical parameters only and compared to current models (21). Some previous good prediction models have been published but have never been used routinely in clinical practice because they are too complicated. Among these, one model considered markers of bone metabolism in 24-h urine collections at different time points besides auxological parameters (42). Stepwise prediction models that had height SDS 0–6 months, height SDS 0–12 months, and growth velocity 0–6 months as outcomes selected both the baseline levels and the change (0–3 months) in the levels of the three miRNAs we validated. Whereas predicted height SDS at 6 months was not significantly improved by the new model with respect to the use of auxological parameters alone; height prediction at 12 months and growth rate at 6 months were highly explained. These latter models also included the GH dose, anticipating the possibility that these models should be further studied for potential use for personalized and optimized treatment. We are aware that the small number of patients could have influenced the findings, and the data need to be confirmed in a much larger dataset; however, we should keep in mind that this type of patient cohort was quite difficult to collect. Further studies are needed to verify whether these miRNAs change or not at puberty, and whether they could be used in growth prediction models in other conditions having GH treatment as an indication. A further use could be hypothesized for the diagnosis of GH deficiency. It is of interest that the value of the information contained in the three miRNA levels and their early variation in predicting growth was greater than that contained in IGF-I serum levels (43, 44), suggesting that miRNAs could effectively be of value for further research.

In conclusion, this exploratory study has shown that miRNAs change on GH treatment, and has led to the identification of three miRNAs that show significant changes in the early period of treatment and contribute to predict the growth response after 12 months. These results are promising and suggest that including miRNAs in the set of variables used to predict treatment response could substantially improve timeliness of correct treatment and contribute to personalized therapy. However, we are aware that these results should be validated in a larger independent cohort of patients and further studies will be required to corroborate miRNA efficacy in the prediction of treatment response. A control group of healthy subjects would also be useful to compare results. In addition, the results suggest that we need to explore these miRNAs as possible identifiers of GH deficiency and finally their potential implication as a cause of idiopathic short stature.

## Data Availability Statement

The original contributions presented in the study are publicly available. These data can be found here: https://www.ncbi.nlm.nih.gov/geo/query/acc.cgi?acc=GSE193450, GSE193450.

## Ethics Statement

This study involving human participants was reviewed and approved by the Ethics Committees of Reggio Emilia and Modena. Written informed consent to participate in this study was provided by the participants’ legal guardian/next of kin.

## Author Contributions

MS conceived, designed, and supervised this study. MES, CS, BR, PL, SP, and BP enrolled the patients. CC, GR, and FC performed the experiments. LB and PGR performed statistical analyses of the data. MES, CC, GR, SA, PGR, LB, and LI contributed to the interpretation of the results. MES, CC, GR, BR, SA, PGR, and LI contributed to writing the manuscript. All the authors read, revised, and approved the final manuscript.

## Funding

This study received funding from the Grant for Growth Innovation (GGI) 2018—Merck. Financial support was also provided by Ipsen S.p.A. and in addition by Fondazione del Monte di Bologna e Ravenna and Ferring S.p.A. The funders were not involved in the study design; collection, analysis, and interpretation of data; the writing of this article; or the decision to submit it for publication.

## Conflict of Interest

The authors declare that the research was conducted in the absence of any commercial or financial relationships that could be construed as a potential conflict of interest.

## Publisher’s Note

All claims expressed in this article are solely those of the authors and do not necessarily represent those of their affiliated organizations, or those of the publisher, the editors and the reviewers. Any product that may be evaluated in this article, or claim that may be made by its manufacturer, is not guaranteed or endorsed by the publisher.
