# Supplementary material for: Specific miRNAs Change After 3 Months of GH treatment and Contribute to Explain the Growth Response After 12 Months
Source: Front Endocrinol (Lausanne). 2022 Jun 22;13:896640. doi: 10.3389/fendo.2022.896640 (PMC9256936; doi:10.3389/fendo.2022.896640)
Supplement: Supplementary file 1 [file Image_1.pdf]

Supplementary Material

Figure S1. miRNA trend over time. MiRNA levels were measured in serum samples from 25 prepubertal patients with IIGHD and are expressed as –ΔCt where ΔCt is calculated as: Ct miRNA - Ct miR-16-5p

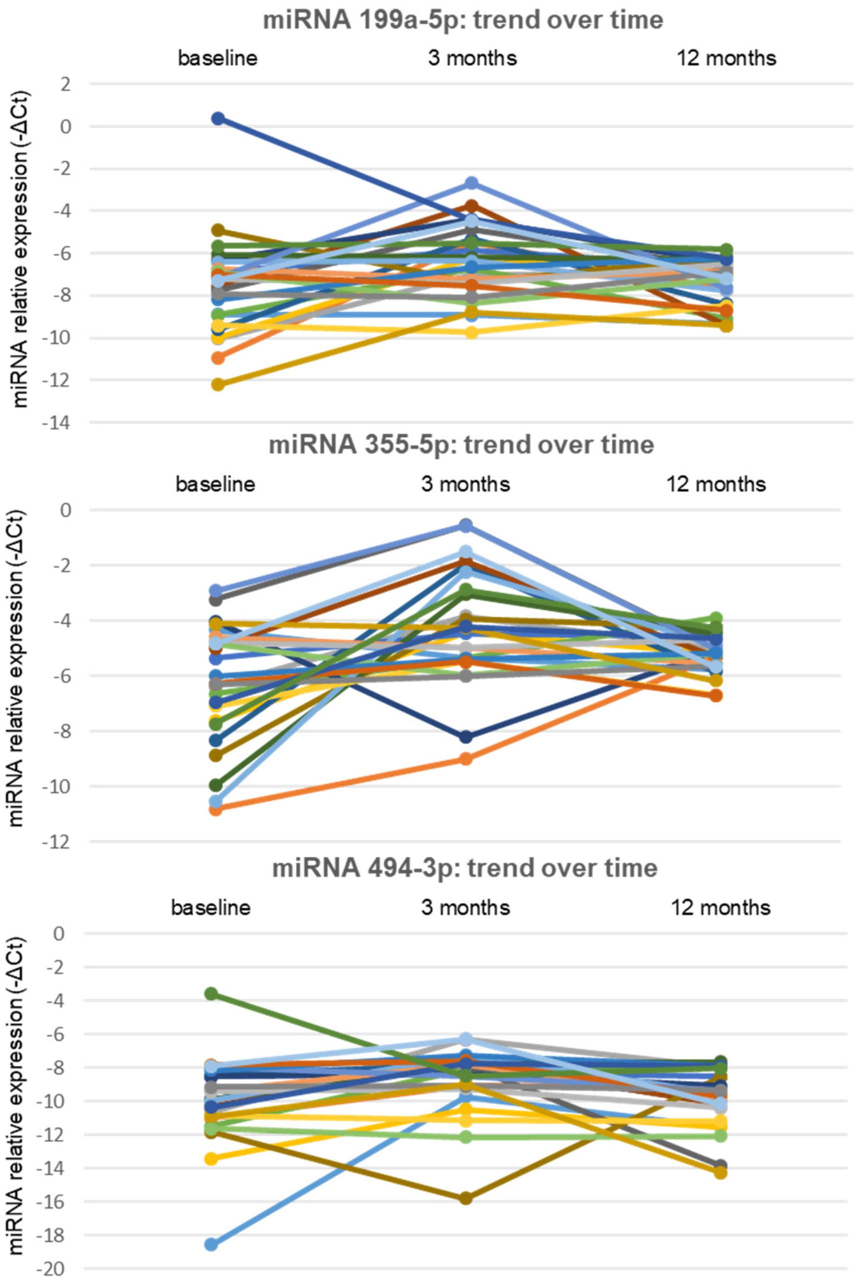

.
